# Supplementary material for: Remapping the cognitive and neural profiles of children who struggle at school
Source: Dev Sci. Author manuscript; Available in PMC 2019 Oct 23. (PMC6808180; doi:10.1111/desc.12747)
Supplement: Supporting Information [file EMS84598-supplement-Supporting_Information.docx]

**Supplementary Material**

*Questionnaire subscales:*

Supplementary Table 1 shows the correlation table for the subscales of the BRIEF.

Supplementary Table 2 shows the correlation table for the subscales of the CCC-2.

Supplementary Table 3 shows the rotated loading matrix for the PCA conducting on the BRIEF subscales, using a varimax rotation.

Supplementary Table 4 shows the rotated loading matrix for the PCA conducted on the CCC-2 subscales, using a varimax rotation.

*Alternative clustering solution*

There is no clear theoretical rationale for how many groups there ought to be within our dataset. The main manuscript includes the profiles that exist when the map is carved into four portions. For information, Supplementary Figure 1 and Supplementary Table 5 include the results if the map is carved into five portions. All clusters from the four-cluster solution have clear analogues in the five-cluster solution and are highly similar.


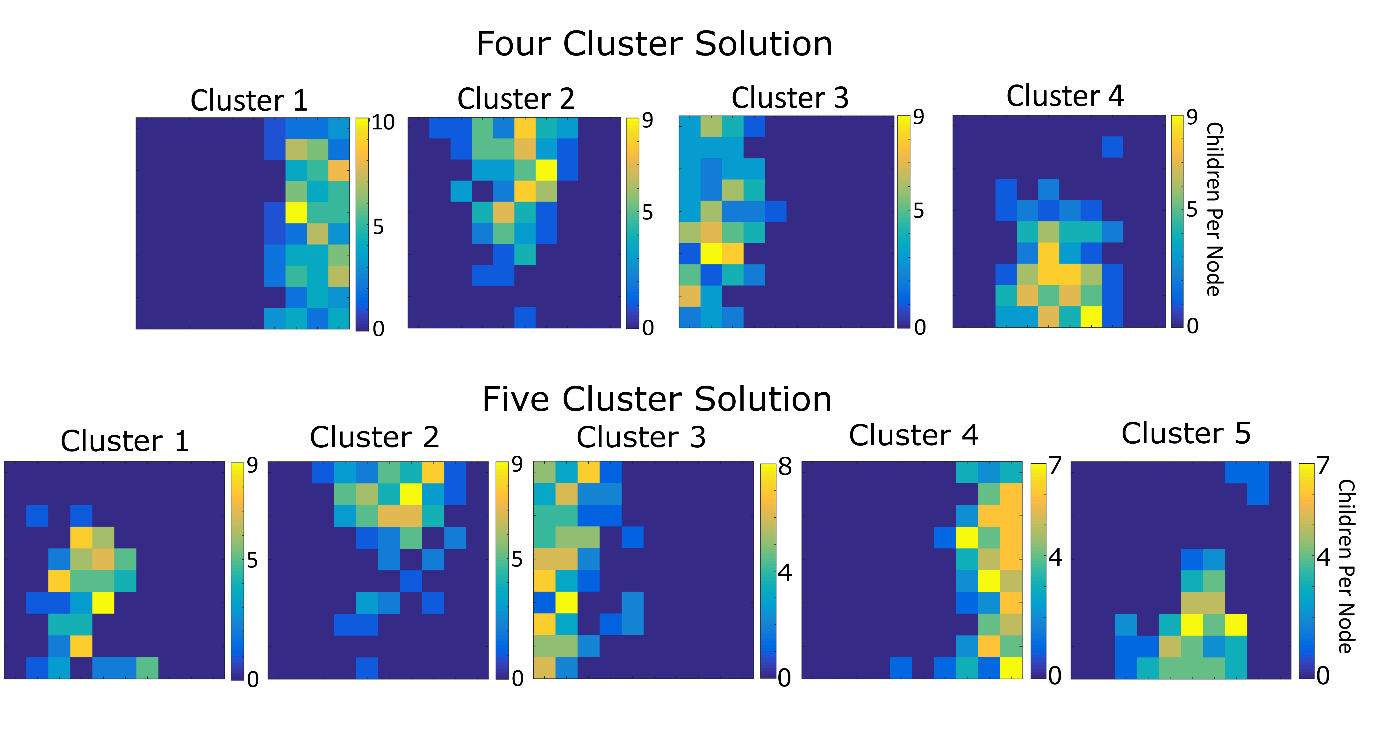
Supplementary Figure 1: Weight maps for a 4- and 5-cluster solution

The groups derived from the five-cluster solution are less distinct on the measures not included in the machine learning. These data can all be seen in Supplementary Table 5. The groups differ significantly in age, although the only significant post-hoc test is between clusters 2 and 5 (p=0.006). Mirroring their counterparts in the four-cluster solution, cluster 3 has significantly more males than we would expect within our sample (Chi Sq=6.490, p=0.0108), and cluster 4 has significantly more females than we would expect for our sample (Chi Squ=9.3622, p=0.0022). The other clusters have the expected ratio.

The profiles of clusters 3, 4 and 5 are very similar to their counterparts in the original four-cluster solution. Here we will focus on clusters 1 and 2, which differ on Vocabulary (p<0.001), Forward Digit Span (p=0.006), Dot Matrix (p<0.001), Backwards Digit (p<0.001) and Mr X (p=0.0182). But their learning profiles are very similar – they do not differ on reading (p=0.1391), or maths (p=0.0940), with only a modest difference in spelling (p=0.0374). Furthermore their behavioural scores do not differ significantly (the difference on the structural language measure is only p=0.1314). On this basis, we favour the four-cluster solution. The additional cluster does not differ substantially on other measures not included in the machine learning. Consequently, it is hard to justify an additional fifth cluster without being confident that it does not reflect overfitting of the cognitive data.

Supplementary Table 5 shows the cognitive, learning and behavioural scores across the five clusters.

*Community detection approach*

An alternative approach to machine learning is community detection using network science. Within network science individual data points (in this case children) are represented as nodes and the correlation between children as edges. The analytic approach explores the extent to which there are boundaries within the data – communities of nodes that are highly correlated with each other, and maximally uncorrelated with members of other communities. To do this, the data must be z scored within the sample before submitting them to the analysis. The extent to which these communities are robust is indicated by a quality index (Q) – the differential between the strength of within group correlations and between group correlations. Submitting our cognitive data to a community detection analysis reveals three groups, but with a Q of only 0.35. This is generally regarded as a poor solution, the distinctiveness and homogeneity of the clusters is not sufficient to justify the separation. If age is regressed from the scores, then the residuals do produce three groups with a more creditable Q=0.46. The separation between these three groups, and their respective z-score profiles can be seen in Supplementary Figure 6.


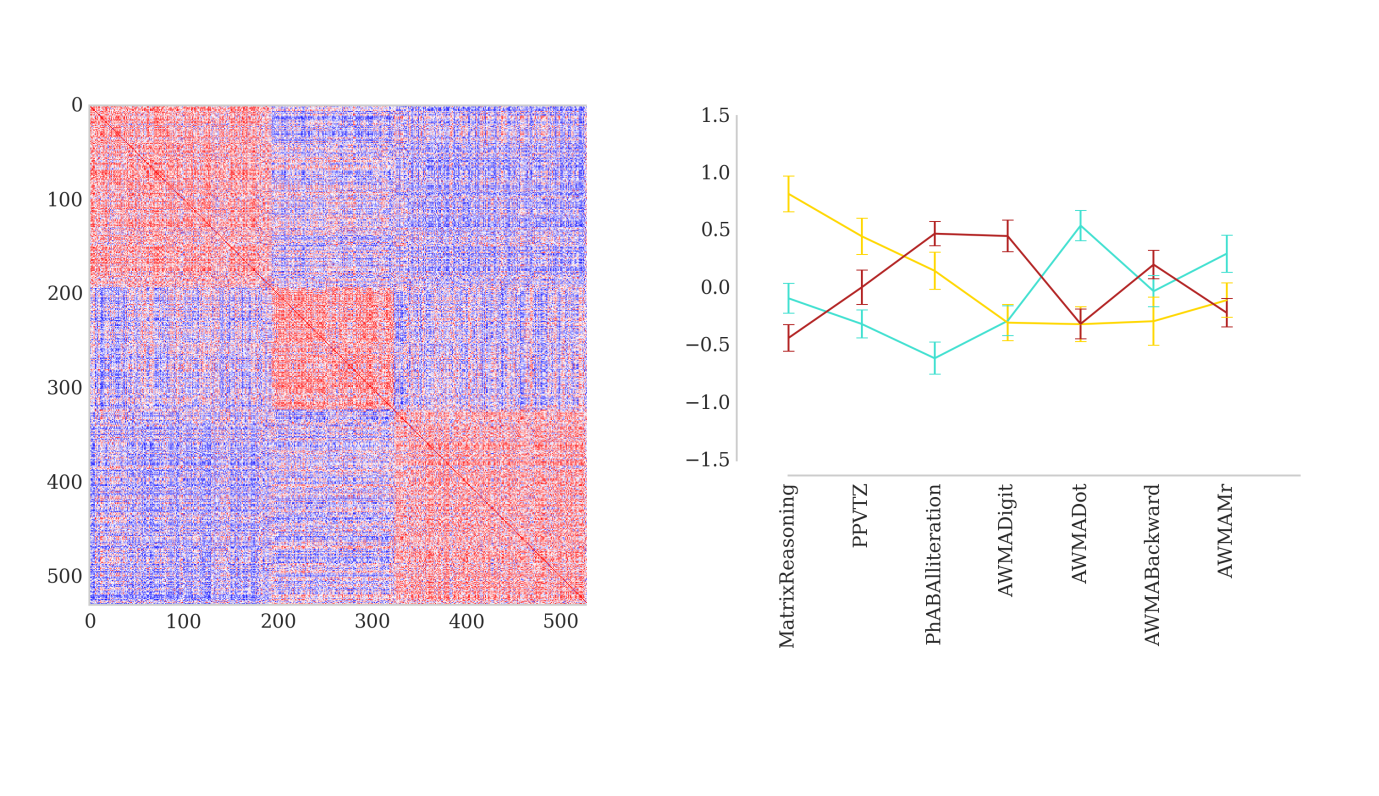


Supplementary Figure 2: The left panel shows the participant-by-participant correlations matrix grouped according to the clustering solution indicated by community clustering. Red colours indicate a positive correlation and blue colours a negative correlation. The right figure panel shows the cognitive profile of the clusters defined through community clustering. The scale represents z-scores of raw performance after regressing age.

The network analysis also distinguishes the two contrasting profiles – children with relative strengths in spatial STM and WM tasks, but deficits in phonological tasks, and children with the converse profile. The network analysis has some strengths over our machine learning approach, more prominently that the number of clusters does not need to be defined, but instead emerges from the data itself. It also produces a statistic (Q) that provides a metric of the quality of the separation. However there are also drawbacks relative to the SOM. Because it is reliant on the correlation between children’s z scores, the community detection is unable to distinguish children with flat profiles. For example, a child with scores of z = 1 on all measures will likely be clustered alongside a child with z = -1 on all measures, because the profile is highly correlated. By contrast, the SOM is able to distinguish these profiles because its main mechanism for clustering is Euclidean distance, and not correlation per se. With the current dataset it is important to distinguish these different profiles.
